# Impact of a hybrid exercise intervention on subjective happiness and self-perceived health of workers: a case study

**DOI:** 10.3389/fspor.2025.1569455

**Published:** 2025-09-05

**Authors:** José M. Núñez-Sánchez, Carmen Sarah Einsle, Jorge López-Fernández, Víctor Jiménez Díaz-Benito

**Affiliations:** ^1^ Faculty of Sports Sciences, Real Madrid Graduate School, Universidad Europea de Madrid, Madrid, Spain; ^2^ Economics and Business Administration Department, Faculty of Economics and Business, University of Málaga, Málaga, Spain; ^3^ Department of Real Madrid Graduate School, Faculty of Medicine, Health and Sports, Universidad Europea de Madrid, Madrid, Spain

**Keywords:** subjective happiness, worker well-being, physical activity, hybrid exercise interventions, self-perceived health, small and medium enterprises (SMEs)

## Abstract

**Introduction:**

Physical activity-based workplace wellness programs (WPPA) are effective in improving health, well-being, and productivity among workers. Despite this, smaller organizations often face challenges in implementing these initiatives. This study assesses the effects of a structured hybrid exercise program, delivered by fitness centers, on subjective happiness, self-perceived health, and physical metrics in Spanish workers during the post-COVID period.

**Methods:**

A pilot study was conducted over three months with 44 participants (45.5% male, 54.5% female) who were sedentary or overweight. The program included a combination of online and in-person exercise sessions. Pre- and post-intervention data were collected using validated tools, such as the SF-36 for self-perceived health and the Subjective Happiness Scale. Statistical methods included paired *t*-tests and multivariate analyses.

**Results:**

The intervention led to significant improvements in subjective happiness (*p* = 0.034; *d* = 0.33) and several dimensions of self-perceived health, including vitality and general health (*p* < 0.01; *d* > 0.50). Overall physical and mental health scores showed moderate enhancements, reflecting the program's effectiveness.

**Discussion:**

The findings demonstrate the potential of hybrid exercise interventions as a practical solution for promoting well-being and happiness in small and medium-sized enterprises that cannot afford to build workplace gyms.

## 1 Introduction

As modern workplaces become increasingly fast-paced and sedentary lifestyles continue to impact employee health and performance, organizations are turning to physical activity-based workplace wellness programs (WPPA) as a proactive solution. These initiatives have proven effective in enhancing not only workers' physical and mental well-being but also their engagement and productivity, ultimately contributing to a healthier and more efficient workforce. Furthermore, strategies and policies that foster employee well-being significantly influences corporate results (1).

The implementation of wellness programs (WPs) to target workers' modifiable health risk behaviors (e.g., smoking, poor nutrition, physical inactivity, etc.), physical and psychological health (e.g., stress, burnout, metabolic syndrome, cardiovascular health, etc.), or productivity and absenteeism is not new (2–6). Among the WPs those based on physical activity (WPPA) are increasing in interest in the literature because they have been evidenced to be effective to improve all these areas: i.e., workers' health risk behaviors, physical and psychological health, and productivity and absenteeism (7, 8). Moreover, implementing WPPA can also result in economic benefits for organizations (9).

Additionally, WPPA are aligned to the UNESCO's Agenda 2030 [Sustainability Objectives 3 and 8 (10)] and have been identified as a key factor to achieve more active societies worldwide [World Health Organization’s 2018 Global Action Plan for PA; Action 2.5; Action 3.3 (11)]. Thus, from the business perspective, WWPPA can be implemented as part of the organization's Corporate Sustainability Responsibility which may help to improve talent acquisition and retention as well as to meet the European Corporate Sustainability Reporting Directive (CSRD) (12–14).

Despite the wide literature supporting the use of WPPA (8, 15) there are some limitations in current literature that need to be addressed. One of them is the fact most research papers are focused on populations from middle or large public or private organizations such as university workers, office workers, industrial and manufacturing workers, civil servants or health-related workers (8, 16), being limited the literature targeting workers from smaller business (17) in which implementing WWPPA may be harder. Another limitation is that, although enhancing PA among workers seems to increase self-perceived happiness and self-perceived health (18), to the authors' knowledge these variables are not very explore in the literature, while the implementation of WWPPA in a company does not mean workers health will improve (19). A third limitation is the large heterogeneity in existing WPPA published probably due to the necessity of individualized each intervention to each study case (8, 15). Moreover, a fourth limitation is that not all organizations own the resources or know-how to implement this kind of program on their own or are just interested in providing a PA program to their workers.

Enhancing workers' PA through a partnership with a fitness center or similar (e.g., leisure centers, boutiques, community centers for exercising, etc.) may be a solution to those cases in which workers are not provided with PA at the workplace as enhancing leisure PA among workers is associated with several health-related benefits (45). This strategy is interesting since fitness centers are designed to support adults to be active (20) and are widely used either for men or women to be active (21), so the workers can engage in leisure PA while the company covers the membership fee. However, although members of fitness centers are more active than non-members (21), careful is need as having free the membership fee may but also may not increase people's current PA levels in all cases (22, 23). Furthermore, the effectiveness of participation of a structure PA program delivered by a fitness center on workers' perceived happiness or health is still unknown. Accordingly, the aim of this study to determine the effect of six-months structured WPPA delivered in workers' leisure time by fitness centers on, subjective happiness and self-perceived health, as well as on the physical and anthropometric parameters of Spanish workers whose organizations were not offering them an occupational WPPA.

This research expects to address the previously reported gap in the literature as well as validate a program that can be implemented by small or middle companies who do not own the resources or know how to implement WWPPA.

## 2 Materials and methods

### 2.1 Study design

This is a pilot study using a single group that voluntarily participated in a three-months hybrid structured exercise program (online and in-person exercise) free of cost delivered through six Spanish fitness centers from the same firm. The research was conducted during post-covid restrictions in Spain, including the obligation to wear a mask in indoor spaces.

### 2.2 Sample

One-hundred-fifty non-clinical workers adults (<18 years old) from different regions of Spain were invited to voluntarily participate in this study. In order to participate in this study candidates (1) had to be in active work environment in a company who is not providing them a WWPPA; (2) could not meet the WHO guidelines for PA through the IPAQ; (3) had to be involved in sedentary occupational activities; (4) had to be overweight; (5) could not being pregnant; and (6) had to telework for at least 3 days/week.

One hundred-thirty-four candidates met the inclusion criteria workers, but only 69% of them completed the initial assessments and signed the consent informed. Apart from this, 10 adults quit before starting the program and 19 quit in the first week. Of the remaining 68 adults, 25 did not meet the criteria of attending at least 80% of sessions and were removed from the study. In total, 43 adults completed ≥80% of the program and both initial and final evaluations.

### 2.3 Procedure

The training program was conducted outside of working hours, classifying it as a leisure-time physical activity intervention, a category that has been associated with greater health and well-being benefits. To ensure inclusivity and facilitate participation, the program was offered at no cost to employees. Additionally, exercise routines were individually tailored, allowing for a personalized approach that considered the specific needs and circumstances of each participant.

Each participant was assigned a personal trainer who provided continuous guidance and oversight throughout the intervention. The process commenced with a comprehensive initial assessment, during which the trainer gathered detailed information on the participant's lifestyle, fitness level, and specific objectives. Based on this assessment, a customized training regimen was developed and administered through a fitness application, ensuring alignment with the participant's individual requirements.

To optimize effectiveness and adherence, the training plan was reviewed and updated on a weekly basis. Brief one-on-one meetings between the participant and trainer, lasting approximately 15 min, informed these adjustments. These sessions served multiple functions: they facilitated progress evaluation, allowed for modifications to the exercise program, and provided a platform to address any challenges encountered by the participant. Moreover, these meetings played a crucial role in maintaining motivation and engagement, reinforcing commitment to the individualized fitness goals established at the outset of the program.

### 2.4 Variables

In order to evaluate the effectiveness of the program and ensure the achievement of the predefined objectives, rigorously validated measurement instruments have been utilized, as detailed in the following Table 1.

**Table 1:** Variables.

Dimension	Instrument	Explanation	Reference
Quality of life	SF-36	Health questionnaire that measures eight health-related dimensions of quality of life such as physical functioning, physical roles, pain, social functioning, emotional roles, mental health, energy and vitality.	Wang et al. (24)
Happiness	Subjective Happiness Scale	To measure happiness developed, to globally evaluate if a person is happy or unhappy, the Subjective Happiness Scale represents a wider classification of wellbeing, measured through global self-assessments.	Lyubomirsky and Lepper (46)

### 2.5 Data analysis

The analyses were conducted using the IBM SPSS Statistics 27.0 software package (IBM Corp., Armonk, NY, USA). Initially, goodness-of-fit tests were performed using the Kolmogorov–Smirnov technique (*N* > 30). A paired-samples *t*-test was conducted to assess the effect of the program between pre- and post-test within the group, independently of the training center attended by the participants. As a measure of effect size, Cohen's d (25) was used for the *t*-tests. The values 0.20, 0.60, and 0.80 were used to interpret the effect size as small, medium, and large, respectively (25). The multivariable analysis was completed using multivariate analysis of variance (ANOVA) for both between-subjects and within-subjects factors (sex: male vs. female, measurement time: pre vs. post, and training center). Pillai's trace was used as the multivariate significance test (26). *post hoc* multiple comparisons were carried out using the Bonferroni technique. When necessary, data were presented in tables including the mean and standard deviation, along with descriptive statistics. Similarly, figures were provided to illustrate the changes, with error bars (95% CI). The effect size was calculated using eta squared (*η*^2^) (*η*^2^ = *Z*^2^/N, where N is the number of observations), and the values 0.01, 0.06, and 0.14 were used to interpret the effect size as small, medium, and large, respectively (25). The significance level was set at 0.05 for all tests.

## 3 Results

Table 2 shows the sociodemographic characteristics of the sample. The mean age of the participants was 39.33 ± 11.49 years. Of these, 51.90% were single and 60.60% were employed. Finally, both pre- and post-test data were obtained from 58 subjects (24 males, 34 females) for the physical and anthropometric parameters, and 43 subjects (20 males, 23 females) for the instruments administered.

**Table 2:** Sociodemographic characteristics of the sample.

Sociodemographic variables	Descriptive Statistics	
Sex *N* (%)		
Men	20	(46.5)
Women	23	(53.5)
Club *N* (%)		
Huelva	9	(20.9)
Granada	5	(11.6)
Málaga	5	(11.6)
Madrid 1	7	(16.3)
Madrid 2	10	(23.3)
Madrid 3	7	(16.3)

M, mean; SD, standard deviation; *N*, sample size, number of subjects.

### 3.1 Analysis of the effect of the within-group intervention on the parameters of subjective happiness, and self-perceived health

Table 3 shows the effect of the intervention on the parameters of physical activity levels, subjective happiness, and self-perceived health. A significant increase in physical activity levels, measured by walking, was observed with a small effect size (*t*_42_ = 2.90; *p* = 0.006; *d* = 0.44), along with an improvement in subjective happiness (item 4) of the instrument (*t*^42^ = 2.19; *p* = 0.034; *d* = 0.33). Scores for the physical function and general health dimensions were significantly higher after the intervention, with a medium effect size (*t*_42_ = 4.59; *p* < 0.001; *d* = 0.70 and *t*_42_ = 0.34; *p* = 0.001; *d* = 0.58). Statistically significant differences were also found in the change from pre- to post-test in the variables of vitality, social function, emotional role, and mental health (*p* < 0.05), as well as in the global components of the scale, with effect sizes (d) above 0.30 in all of them.

**Table 3:** Analysis of the effect of the within-group intervention on the parameters of physical activity levels, subjective happiness, and self-perceived health.

	Pre-test (*N* = 43)		Post-test (*N* = 43)				IC 95%		
	M	SD	M	SD	t	*p*	d	Lower	Upper
Happiness (Subjective Happiness Scale)									
Item 1	5.56	1.40	5.77	1.23	1.10	0.277	0.17	−0.47	0.13
Item 2	5.74	1.16	5.81	1.14	0.46	0.645	0.07	−0.37	0.23
Item 3	5.21	1.41	5.51	1.37	1.87	0.068	0.29	−0.59	0.02
Item 4	4.60	1.98	5.23	1.57	2.19	0.034*	0.33	−0.64	−0.03
Self-perceived health (SF-36)									
Physical function (0–100)	91.74	10.74	96.98	5.47	4.59	<.001***	0.70	−1.03	−0.36
Physical role (0–100)	23.40	4.94	24.13	3.22	0.87	0.39	0.13	−0.43	0.17
Body pain (0–100)	88.60	14.45	89.44	12.21	0.34	0.735	0.05	−0.35	0.25
General health (0–100)	69.21	18.96	78.51	13.62	3.79	<.001***	0.58	−0.90	−0.25
Vitality (0–100)	57.44	10.88	63.02	12.11	3.17	0.003**	0.48	−0.80	−0.16
Social function (0–100)	82.27	25.91	91.28	19.96	2.10	0.042*	0.32	−0.63	−0.01
Emotional role (0–100)	19.57	9.07	22.87	5.48	2.65	0.011*	0.40	−0.71	−0.09
Mental health (0–100)	64.19	13.27	68.65	11.34	2.78	0.008**	0.43	−0.73	−0.11
Physical health (global) (0–100)	52.67	3.60	54.39	2.58	3.36	0.002**	0.51	−0.83	−0.19
Mental health (global) (0–100)	35.53	7.17	38.12	6.03	2.42	0.02*	0.37	−0.68	−0.06

Values of 0.20, 0.60, and 0.80 were used to interpret the effect size as small, medium, and large (25). Significance level at *p <  0.05; **p < 0.01; ***p < 0.001. M, mean; SD, standard deviation; t, student's *t*-test; CI, 95% confidence interval; d, Cohen's d.

### 3.2 Analysis of the effect of the intervention according to the sports center on the parameters of subjective happiness and self-perceived health

Significant differences were observed in item 4 of the subjective happiness scale with a medium effect size (F1,37 = 6.15; *p* = 0.034; *η*p2 = 0.14), although no significant differences were found in the *post hoc* multiple comparisons (Supplementary Figure S1). The global component of both the physical health scale (F1,37 = 12.61; *p* = 0.01; *η*p^2^ = 0.25) and mental health (F1,37 = 7.09; *p* = 0.011; *η*p2 = 0.16) were significantly improved, with a large effect size in the within-group analysis. However, no significant differences were found in the interaction with the training center or in the *post hoc* multiple comparisons by center (*p* > 0.05) (Supplementary Figure S2).

### 3.3 Analysis of the effect of the intervention according to sex on subjective happiness and self-perceived health

After the intervention, men scored statistically higher than women on item 4 of the subjective happiness scale, which describes the characterization of unhappy people (F1.41 = 481; *p* = 0.034; *η*p2 = 0.11). There was no interaction in this variable according to the sex of the participant (*p* > 0.05) (Supplementary Figure S3). Intrasubject analysis showed a statistically significant improvement in the global component of the physical and mental health scale, (F1.41 = 10.91; *p* = 0.02; *η*p2 = 0.21 y F1.41 = 5.95; *p* = 0.19; *η*p2 = 0.13 respectively), although the interaction with the sex of the participants was not significant (*p* > 0.05) (Supplementary Figure S4).

## 4 Discussion and conclusion

The main objective of the present study was to determine the effect of a structured WPPA delivered by different sports centers on the parameters of subjective happiness and self-perceived health, as well as on the physical and anthropometric parameters of Spanish workers, whose organizations were not offering them an occupational WPPA.

The benefits of regular physical activity and exercise for health and well-being (27, 28) and the benefits of the implementation of WPPA in the workplace have been widely discussed in literature. These benefits of WPPA also were highlighted in the present study but implementing such programs within small and medium sized companies poses unique challenges. These smaller enterprises often face limitations such as the lack of expertise, space, resources, prohibitive costs, and personnel or time constraints to implement a WPPA in their workplaces (29).

Given these constraints, alternative strategies are necessary to promote happiness and well-being within SMEs. A viable solution is the promotion of workers' physical activity through partnerships with fitness centers, as shown in the current intervention. This program was conducted outside working hours, personalized, and free of charge for participants. While increasing physical activity during working hours may not be feasible, incentivizing PA during leisure time can effectively mitigate these limitations. For instance, companies could subsidize membership fees for workers, enabling them to access fitness centers. By covering membership fees, companies can encourage employees to engage in leisure PA, which has been shown to yield various health-related benefits (45). However, it is important to recognize that providing free membership fees does not necessarily increase PA levels (22, 23).

Although numerous studies focused on supervised physical exercise interventions for health improvement or disease treatment (30), less frequently studied parameters, i.e., self-perceived happiness and self-perceived health (18), were rarely analyzed. Thus, not only possible improvements in physical activity variables as such, but also aspects related to self-perceived health, and the state of subjective happiness were analyzed in the present study.

Regarding happiness, improvements were found across all four items from pre- to post-test, with statistically significant changes in item 4, although these were not upheld in *post hoc* comparisons. No significant gender differences were found. Barsasella et al. (31) demonstrated that augmented reality exercise interventions in elderly populations could significantly increase happiness and improve anxiety and depression levels. While our study did not yield similarly pronounced results in the happiness domain, it underscores the potential for structured interventions to elicit measurable psychological benefits. Similarly, the results of a meta-analysis on overweight or obese adults showed that physical exercise *per se* does not usually have a significant effect on reducing depression, large effects on improving physical quality of life and to a lesser extent on vitality and mental health were observed (32). Also in previous studies, supervised exercise programs showed effects on the mental factor of work capacity (33), as well as the benefits of physical activity for the improvement of mental health, social aspects in adults and quality of life were confirmed (32, 34). These findings emphasize the multifaceted benefits of PA, though they also reveal the complexity of achieving consistent improvements across all psychological dimensions. The emphasis on enhancing psychosocial variables is not a common feature in comparable academic studies, positioning this approach as a novel contribution that addresses a significant gap in the existing literature.

It is important to note that the intervention was conducted between April and August 2021, during the post-COVID recovery phase. Participants were still affected by residual restrictions and psychological stressors tied to the pandemic. In the post-COVID period, studies showed lower levels of physical activity among female university students with corresponding negative impacts on well-being compared to their male counterparts (35, 36). In contrast, in the present study, considering the subjective happiness and self-perceived health, the intrasubject analysis revealed a statistically significant improvement in the overall component of the physical and mental health scale, although the interaction with the sex of the participants was not significant. However, the results regarding the effects of the intervention according to sex differ in the literature. For example, in a workplace health promotion program, women's psychological anxiety decreased over time, but this change was only partially attributed to participation in the program, and only to lifestyle interventions, while men's did not change over time (37). In contrast, in an intervention in children, boys moved significantly more than girls did (38).

The duration of interventions must also be carefully controlled. Authors such as Corres et al. (39) emphasize the need for regular, supervised programs to maintain achieved improvements. Although our study confirmed statistically significant improvements after almost five months of intervention, another study found that a four-month program was not enough to affect work capacity, which improved after 12 months (40). However, other studies also showed significant effects on different parameters in shorter interventions ≤20 weeks (41–43). Another limitation of this research is the absence of a control group, due to budget constraints and the voluntary nature of participant recruitment. This limitation was driven by the company's focus on piloting feasibility rather than implementing a controlled design.

Improving participant retention and adherence remains a key priority in workplace physical activity interventions. In our study, schedule incompatibility emerged as a major factor contributing to dropout, as frequently reported in systematic reviews and meta-analyses. This challenge significantly contributes to the typical 20% attrition rate observed in workplace wellness programs (7, 44). Participants who complete such programs tend to exhibit higher baseline motivation and fitness levels, while the absence of tangible incentives may further discourage sustained engagement. These elements may intersect with gender disparities, as women often face additional barriers to maintaining consistent physical activity routines. Methodologically, this underscores the importance of intention-to-treat analyses and of scrutinizing attrition patterns to determine whether dropout is random or systematically linked to participant characteristics. Although our intervention yielded significant adherence-related improvement variables, future studies should incorporate more robust research designs to avoid attributing outcomes to chance. Integrating evidence-based behavioral theories into intervention planning could also enhance retention, though the literature in this area continues to evolve.

To address this issue, drawing on our findings and existing literature, we propose four practical measures. First, administer brief barrier-access questionnaires to identify potential challenges early on. Second, prioritize participants with compatible schedules through coordination with departments. Third, establish gradual withdrawal protocols to reduce abrupt dropouts. Fourth, implement accountability partnerships to promote mutual motivation. Additionally, grounding interventions in established physical activity promotion models may improve adherence, although this remains an evolving area. Future research should explore how hybrid interventions can be adapted to fit diverse organizational and cultural contexts.

Additionally, anchoring interventions in established physical activity promotion frameworks, such as the model proposed by Núñez-Sánchez et al. (35, 36), may further improve adherence. These authors propose a well-being management framework, based on a successful experience, offering a proven, modular and flexible framework model for all kind of companies. Future studies should investigate the adaptability of hybrid intervention models to diverse cultural and organizational contexts.

### 4.1 Conclusion

This pilot study demonstrates that hybrid physical activity programs, delivered in collaboration with external fitness centers, can produce significant improvements in physical activity levels, subjective happiness, and self-perceived health among employees. For SMEs without on-site resources or infrastructure, this model represents a feasible and promising alternative.

While our intervention proved financially viable in this specific case, future research should explore whether these health benefits correlate with measurable organizational outcomes, such as enhanced productivity, retention, or employee engagement. Scaling up such programs will likely depend on both evidence-based support and organizational culture, along with socio-labor dynamics. In this respect, frameworks like.

In summary, this study contributes to filling the gap of case studies on workplace health and well-being promotion in SMEs by offering a replicable blueprint for SME settings. The documented improvements in happiness and health reaffirm the value of promoting leisure-based physical activity, even when direct implementation within the workplace is not feasible. Despite methodological limitations, our intervention provides a practical and innovative approach for future refinement within occupational health and well-being strategies.

## Data Availability

The raw data supporting the conclusions of this article will be made available by the authors, without undue reservation.
