# Supplementary material for: Impact of a hybrid exercise intervention on subjective happiness and self-perceived health of workers: a case study
Source: Front Sports Act Living. 2025 Sep 5;7:1569455. doi: 10.3389/fspor.2025.1569455 (PMC12446260; doi:10.3389/fspor.2025.1569455)

Supplementary Material

## Supplementary Figures

**Supplementary Figure 1.** Analysis of the effect of the intervention by Training Center on the parameters of subjective happiness


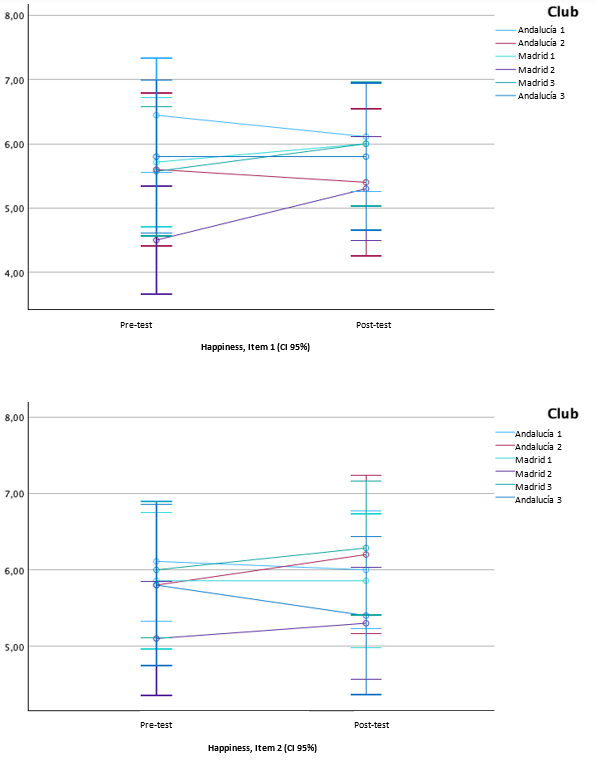


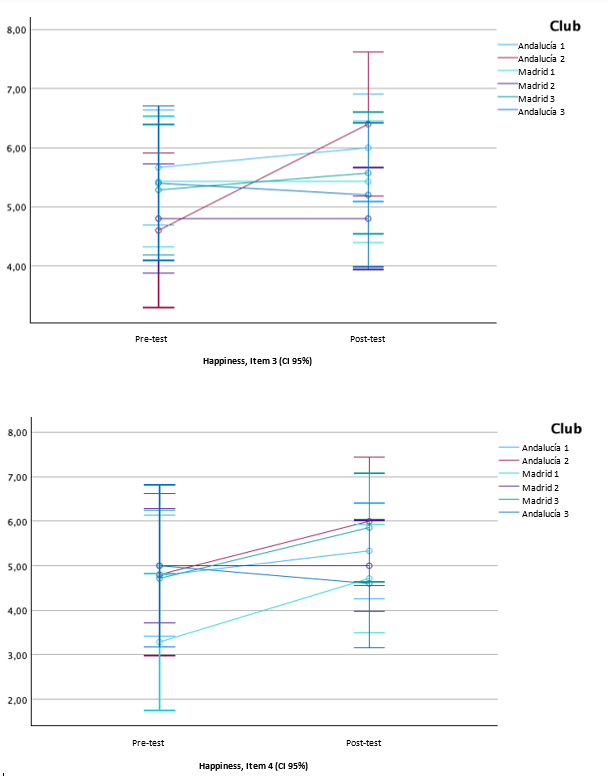


**Supplementary Figure 2.** Analysis of the effect of the intervention according to the Sports Center on self-perceived health parameters


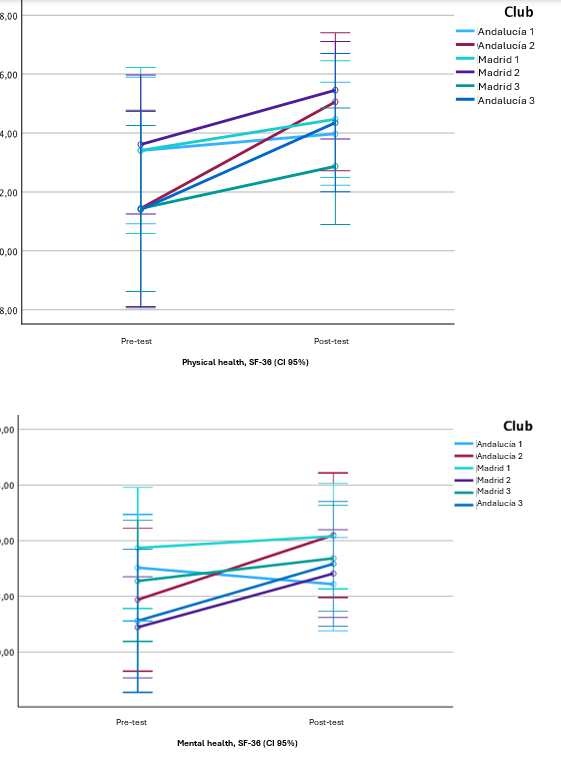


**Supplementary Figure 3.** Analysis of the effect of the intervention according to sex on the parameters of subjective happiness levels

**Supplementary Figure 4.** Analysis of the effect of the intervention according to sex on self-perceived health parameters


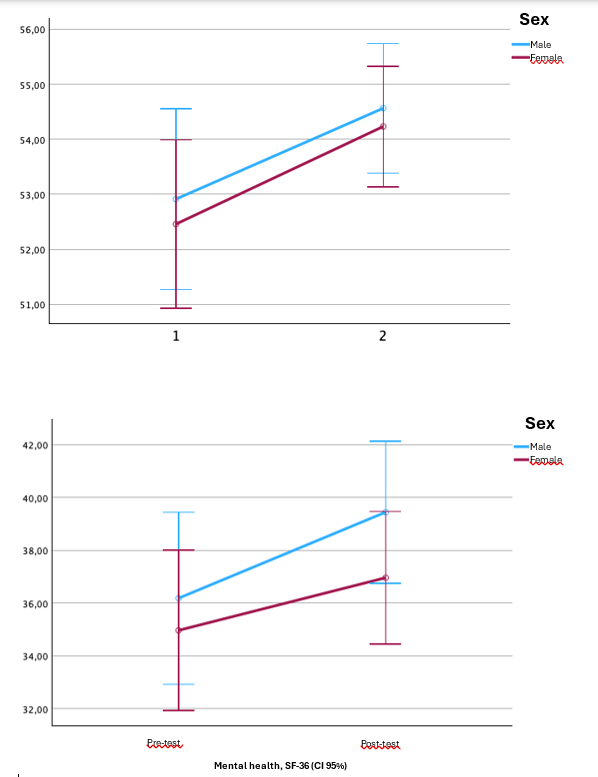

Supplement: Supplementary file 1 [file Table1.docx]
